# Maintenance of Blood-Brain Barrier Integrity in Hypertension: A Novel Benefit of Exercise Training for Autonomic Control

**DOI:** 10.3389/fphys.2017.01048

**Published:** 2017-12-12

**Authors:** Leila Buttler, Maria T. Jordão, Matheus G. Fragas, Adriana Ruggeri, Alexandre Ceroni, Lisete C. Michelini

**Affiliations:** Department Physiology and Biophysics, Institute of Biomedical Sciences, University of São Paulo, São Paulo, Brazil

**Keywords:** spontaneously hypertensive rats, blood-brain barrier, autonomic control, exercise training, angiotensin II, microglia, hypothalamus, brain stem

## Abstract

The blood-brain barrier (BBB) is a complex multicellular structure acting as selective barrier controlling the transport of substances between these compartments. Accumulating evidence has shown that chronic hypertension is accompanied by BBB dysfunction, deficient local perfusion and plasma angiotensin II (Ang II) access into the parenchyma of brain areas related to autonomic circulatory control. Knowing that spontaneously hypertensive rats (SHR) exhibit deficient autonomic control and brain Ang II hyperactivity and that exercise training is highly effective in correcting both, we hypothesized that training, by reducing Ang II content, could improve BBB function within autonomic brain areas of the SHR. After confirming the absence of BBB lesion in the pre-hypertensive SHR, but marked fluorescein isothiocyanate dextran (FITC, 10 kD) leakage into the brain parenchyma of the hypothalamic paraventricular nucleus (PVN), nucleus of the solitary tract, and rostral ventrolateral medulla during the established phase of hypertension, adult SHR, and age-matched WKY were submitted to a treadmill training (T) or kept sedentary (S) for 8 weeks. The robust FITC leakage within autonomic areas of the SHR-S was largely reduced and almost normalized since the 2nd week of training (T_2_). BBB leakage reduction occurred simultaneously and showed strong correlations with both decreased LF/HF ratio to the heart and reduced vasomotor sympathetic activity (power spectral analysis), these effects preceding the appearance of resting bradycardia (T_4_) and partial pressure fall (T_8_). In other groups of SHR-T simultaneously infused with *icv* Ang II or saline (osmotic mini-pumps connected to a lateral ventricle cannula) we proved that decreased local availability of this peptide and reduced microglia activation (IBA1 staining) are crucial mechanisms conditioning the restoration of BBB integrity. Our data also revealed that Ang II-induced BBB lesion was faster within the PVN (T_2_), suggesting the prominent role of this nucleus in driven hypertension-induced deficits. These original set of data suggest that reduced local Ang II content (and decreased activation of its downstream pathways) is an essential and early-activated mechanism to maintain BBB integrity in trained SHR and uncovers a novel beneficial effect of exercise training to improve autonomic control even in the presence of hypertension.

## Introduction

Chronic hypertension is a major risk factor for coronary artery disease, ischemic and hemorrhagic stroke, congestive heart failure, peripheral vascular, and other diseases (James et al., 2014). Accumulating experimental and clinical evidence has shown that neurohormonal control of the circulation is impaired in hypertensive individuals. Circulatory control in hypertension is characterized by baroreflex dysfunction and autonomic dysregulation, leading to sympathetic overactivity, activation of plasma and tissue renin-angiotensin system (RAS) and of circulating catecholamines. Increased angiotensin II (Ang II) availability activates intracellular pathways and increases neuronal activity within the autonomic areas as the hypothalamic paraventricular nucleus (PVN) and brainstem nuclei (nucleus of the solitary tract, NTS; rostral ventrolateral medulla, RVLM) that have been shown to contribute to neurogenic hypertension (Kang et al., 2009; Shi et al., 2010; Waki et al., 2011; Zubcevic et al., 2011). Indeed, both circulating Ang II (via AT_1_ receptors in endothelial cells) and locally synthetized Ang II cause vascular dysfunction and microglia activation, increase the production of reactive oxygen species, eNOS uncoupling and augment pro-inflammatory cytokines synthesis, which are important causal factors for sympathoexcitation in neurogenic hypertension (Shi et al., 2010; Waki et al., 2011; Zubcevic et al., 2011).

Recent studies have suggested another mechanism by which circulating Ang II gains access to autonomic areas inside the blood-brain barrier (BBB). An increased BBB permeability allowing the entry of circulating Ang II within the brain parenchyma of the PVN, RVLM, and NTS was documented in renal and spontaneously hypertensive rats (SHR) (Biancardi et al., 2014; Biancardi and Stern, 2016). Within the PVN Ang II interacts with AT1 and Toll-like receptor 4, activates microglial cells, increases local oxidative stress and causes sympathoexcitation (Biancardi et al., 2016). BBB disruption not only facilitates Ang II access, but also allows circulating inflammatory cells to enter into the brain parenchyma, contributing to further microglial activation and inflammation in autonomic areas (Waki et al., 2011; Zubcevic et al., 2011). These responses alter neurovascular coupling, dysregulate cerebral perfusion and markedly augment neuronal discharge, thus exacerbating the sympatho-humoral activation in hypertensive animals (de Vries et al., 1997; Zhang et al., 2010). Loss of BBB integrity within the cortex and hippocampus with dysregulation of local perfusion was also observed in neurodegenerative diseases including Parkinson and Alzheimer, stroke, traumatic brain injury, and other disorders (Rosenberg, 2012; Erickson and Banks, 2013; Schoknecht et al., 2015).

The search for therapies to improve BBB integrity and ameliorate the autonomic control of the circulation has been pursued by several researchers and physicians. The reduction of BBB permeability within the PVN, RVLM, and NTS of hypertensive rats treated with AT_1_ receptors blockade (Biancardi et al., 2014; Biancardi and Stern, 2016) highlights the importance of RAS inhibition. Besides the autonomic areas, blockade of AT_1_ receptors has also shown to reduce BBB permeability in the hippocampus and cortex (Kucuk et al., 2002; Pelisch et al., 2011). Although AT_1_ receptor blockade was accompanied by neuroprotective effects in cognitive disorders (Pelisch et al., 2011) and blood pressure reduction (Kucuk et al., 2002; Biancardi et al., 2014), there is no information on autonomic control following normalization of BBB integrity. Moreover, it was reported recently that candesartan and ursodeoxycholic acid reduced blood pressure in obese mice but did not prevent BBB dysfunction and cognitive deficits (Mamo et al., 2017).

On the other hand, studies from our and other laboratories have reported the efficacy of aerobic training to downregulate brain RAS and correct autonomic dysfunction in SHR (Pan et al., 2007; Agarwal et al., 2011). Trained SHR exhibited an early (2 weeks) and maintained normalization of angiotensinogen expression within autonomic areas, which was correlated to the reduced sympathetic outflow to heart and vessels and preceded the partial pressure fall (Chaar et al., 2015). Trained SHR also showed prompt (2 weeks) normalization of baroreceptor reflex control of heart rate that coincided with a marked reduction of oxidative stress and inflammation in the PVN (Masson et al., 2014). Similar training-induced effects were also observed in other autonomic areas (Pan et al., 2007; Agarwal et al., 2011). Knowing that hypertension is characterized by autonomic impairment, BBB leakage, and Ang II-induced neuronal activation and that exercise training is highly effective to prevent Ang II-induced effects and to improve autonomic control, we hypothesized that training may improve autonomic control by restoring BBB integrity and normalizing brain perfusion in hypertensive individuals. Because there is no information on possible beneficial effects of exercise training on BBB leakage in the SHR (the best model of essential hypertension), we sought to evaluate in autonomic brain areas: (i) the changes on both BBB permeability and circulatory autonomic control induced by training starting in the chronic phase of hypertension; (ii) the combined effect of both training and elevated brain Ang II content on BBB permeability. In addition, because the BBB is completely formed and functionally competent at birth (Hagan and Ben-Zvi, 2015) we undertook a temporal study using sedentary SHR, examining the integrity of the BBB over time (from 1 to 5 months) to determine when the integrity of the BBB within autonomic areas becomes impaired. Normotensive Wistar Kyoto rats (WKY) were used as controls.

## Materials and methods

### Animal surgeries, experimental protocols, and hemodynamic recordings

This study was carried out in compliance with the Ethical Principles in Animal Research of the Brazilian College of Animal Experimentation. The protocols were approved by the Institutional Animal Care and Use Committee of the University of Sao Paulo. Groups of young (20–22 days) and adult (12–13 weeks) male SHR and age-matched WKY were housed in the Animal Facilities of the Department of Physiology and Biophysics under controlled temperature/humidity, 12/12 h light/dark cycle, with free access to water and standard laboratory chow.

SHR and age-matched WKY were submitted to three experimental protocols: (i) Blood pressure measurement and quantification of BBB permeability in autonomic areas of young rats during 5 months life span from the pre-hypertensive up to the chronic phase of hypertension; (ii) Analysis of the temporal effects induced by 8-weeks aerobic exercise training or sedentary protocol on both hemodynamic parameters and BBB permeability in brain areas of adult SHR and WKY aged 3 month; (iii) Analysis of the combined effect of training and *icv* Ang II infusion on BBB permeability, AT_1_ receptor expression and microglia density within autonomic areas of the adult SHR. In all these protocols, BBB permeability was examined within the PVN, NTS, and RVLM, important autonomic nuclei controlling sympathetic and parasympathetic outflow to heart and vessels.

#### Protocol I

Young SHR and WKY (50–70 g) were kept sedentary in Plexiglass cages. At the 1st, 2nd, 3rd, 4th, and 5th months chronic catheters (Tygon Tubing, Critchley, Australia: 2 cm of 0.28:0.61 connected to 7 cm of 0.50:1.50 ID:OD) were implanted in the left femoral and left carotid arteries under anesthesia (ketamine, 80 mg/kg plus xylazine, 12 mg/kg, *ip*). They were treated *sc* with analgesic (ketoprofen 1%, 2 mg/kg; Biofarm, Jaboticabal, Brazil) and enrofloxacin (Baytril 5 mg/kg, Bayer Sao Paulo, Brazil) and allowed to recover for 24 h. Time-course changes of resting arterial pressure (AP) were recorded for 30–40 min (computer, 2,000 Hz of sampling frequency, LabChart Pro, ADInstruments Bella Vista, NSW, Australia) on the next day in conscious unrestrained rats, as previously described (Masson et al., 2014; Ichige et al., 2016). At the 1st, 3rd, and 5th month after AP recordings, 10 kDa fluorescein isothiocyanate dextran (FITC, 10 mg/mL, Sigma Aldrich, USA) plus 70 kDa rhodamine B isothiocyanate dextran (RHO, 10 mg/mL Sigma Aldrich, USA), 286 μL/100 g each, were administered at a slow rate (70 and 300 μL/min in rats aged 1 and 3–5 months, respectively) via the carotid artery and allowed to recirculate for 20 min (Biancardi et al., 2014). Rats were then deeply anesthetized (overdose of ketamine + xylazine, 240 mg/kg + 36 mg/kg, *ip*) for brain harvesting immediately after the respiratory arrest. Brains were post-fixed in 4% phosphate-buffered (PB) paraformaldehyde for 48 h, cryoprotected (30% sucrose in 0.01 M PBS) for 72 h at 4°C and stored (−80°C) until processing.

#### Protocol II

During a 2-week acclimatization period, adult SHR and WKY (250–280 g) were preselected for their ability to walk/run on a treadmill (KT-300, Inbramed, Porto Alegre, Brazil; 10 sessions from 0.4 to 0.8 km/h, 0% grade, 10 min/day). Only active rats (those able to walk/run on the treadmill during the adaptation period, corresponding to 98% of the SHR and 93% of the WKY) were included in this protocol. Rats were then subjected to progressive maximal exercise tests (MET, Cavalleri et al., 2011) to determine maximal individual aerobic capacities, whose values were used to allocate rats with identical capacities to trained (T) and sedentary (S) groups and to set the intensity of aerobic exercise training (50–60% of the maximal exercise capacity, performed 5 days/week, 1 h/day for 2 months, Masson et al., 2014). MET were repeated at weeks 4 and 8 of both protocols in order to adjust and maintain the training intensity and compare the protocols' efficacy, respectively. Rats from S groups were handled every day and subject once per week to a short period of mild exercise (5–10 min, 0.4–0.8 km/h, 0% grade) to approximate their conditions to those experienced by T groups (Cavalleri et al., 2011; Masson et al., 2014). At predetermined periods (weeks 0, 1, 2, 4, and 8 of T and S protocols), SHR and WKY were anesthetized (ketamine + xylazine, 80 mg/kg +12 mg/kg, *ip*) for chronic catheterization of the femoral (hemodynamic parameters' recordings) and carotid (FITC + RHO injection) arteries. Similar to young groups, rats were treated with analgesic plus antibiotic *sc* and recovered for 24 h. Baseline AP and heart rate (HR) were continuously acquired on a beat-to-beat basis (30–40 min, LabChart Pro, AD Instruments, sampling frequency of 2,000 Hz) on the next day in conscious freely moving rats resting in their experimental cage (Masson et al., 2014; Ichige et al., 2016). BBB permeability was analyzed after the hemodynamic recordings. The mixture of dyes was administered via the carotid artery and the rats were deeply anesthetized 20 min later (Biancardi et al., 2014); brains were harvested immediately after the respiratory arrest, post-fixed, cryoprotected, and stored until processing.

#### Protocol III

Active adult SHRs were adapted to the treadmill for 2 weeks and submitted to MET. Rats were anesthetized (ketamine, 100 mg/kg + xylazine, 20 mg/kg, *ip*) for the implantation of an infusion kit attached to an osmotic mini-pump within the lateral ventricle (*Brain infusion kit 2* + *Micro-osmotic pump* model 1002, Alzet, Cupertino CA, USA) as previously described (DeVos and Miller, 2013). Briefly, rats were placed in a stereotaxic apparatus (David Kopf, Tujunga CA, USA, incisive bar −3.3 mm) and after local anesthesia (2% lidocaine chloridrate + 0,04% phenylephrine chloridrate) and asepsis (iodate alcohol solution), the skull was exposed and a small hole opened for the insertion of the infusion kit into the lateral ventricle (1.0 caudal to the Bregma, 1.6 mm lateral to the midline, 4.5 mm ventral to the skull surface) (Paxinos and Watson, 2007). The infusion kit was fixed with fast polymerizing methacrylate and connected to the osmotic mini-pump (0.25 μL/h for 14 days) previously filled with saline or angiotensin II (Ang II, 50 ng/μL/h) and inserted subcutaneously in the scapular region. The skin was sutured; the rats were treated with analgesic plus antibiotic and allowed to recover in their individual cages. Exercise training (T = 50–60% of maximal exercise capacity, 1 h/day) or the S protocol started in the next day and continued for another 13 days, during the active infusion period of the mini-pumps. Notice that 14 consecutive daily exercise sessions and training for 2–3 weeks (protocol II) represent a similar volume of training, that is, they are isocaloric. At the end of protocols rats were anesthetized and cannulated. In the following day, after hemodynamic recordings half of these rats were infused with FITC+RHO intra-arterially and brains removed 20 min later for immunofluorescence assays, as described above; in the other half, the brains were quickly perfused with sterile 0.01 M PBS (100 mL, pH 7.4) followed by 4% paraformaldeyde (400 mL, 20–30 mL/min, Daigger pump, Vernon Hills IL, USA). Brains were removed, post-fixed (4 h), cryoprotected (72 h at 4°C), stored (−80°C) until processing and used to analyze the combined effects of training and Ang II infusion on AT1 receptors' expression and microglia activation.

### Power spectral analysis

Time series of resting systolic AP (SAP) and pulse interval (PI) were used to analyze pressure and HR variability, as previously described (Japundzic et al., 1990; Task Force of the European Society of Cardiology, North American Society of Pacing and Electrophysiology, 1996; Eckberg, 1997). Briefly, time- and frequency-domain analyses were evaluated using fast Fourier transformation by Welch's method and Hanning windows with 50% overlap. Power spectral density for low frequency (LF, 0.20–0.75 Hz, indicating mainly the sympathetic activity to vessels and sympathetic + parasympathetic modulation of the heart) and high frequency domain (HF, 0.75–3.0 Hz, indicative of the vagal activity to the heart) were obtained by means of power spectrum density integration within each frequency bandwidth, using a customized routine (MATLAB6.0, Mathworks, Natick, MA, USA. Ceroni et al., 2009; Masson et al., 2014). LF/HF ratio to the heart and the spontaneous baroreflex sensitivity through the αLF and αHF indexes (representing baroreflex sensitivity at the LF and HF bands, respectively, Laude et al., 2004; Martelli et al., 2014) were also assessed.

### BBB leakage analysis

Sequential coronal brain slices (30 μm) containing the PVN, NTS, and RVLM (Paxinos and Watson, 2007) were obtained in the cryostat (Leica CM1850, USA), collected in tissue culture wells with 0.1 M PBS, placed on gelatinized slides and mounted with Slowfade® Gold Antifade Reagent (Life Technologies CA, USA). Tissues were examined on a fluorescence microscope (Leica DMLB, Nussloch, Germany) attached to an ExiBlue camera (Imaging, Canada); the selected images were acquired by the Image-Pro Plus software (v7.01, Media Cybernetics, USA) and quantified by the ImageJ software (NIH, USA). BBB permeability, evaluated by the capability of the small size fluorescent dextran (FITC) to remain within vessels or partially leak into the brain parenchyma in the presence of compromised BBB integrity, was quantified as previously described (Biancardi et al., 2014). Briefly, a binary image containing only the FITC signal (extra + intravascular), was subtracted from a binary image of the colocalized RHO-FITC image (intravascular only). The degree of leaked FITC in the subtracted image was quantified by the proportion of positive pixels in the acquired image; individual results were averaged per group and means compared among experimental groups. To confirm the specificity of BBB permeability within autonomic areas, FITC leakage was also analyzed in the hypoglossus nucleus, which does not participate in autonomic control and is not affected by exercise training.

### Immunofluorescence assays

Similar coronal brain sections interesting the PVN, NTS, and RVLM were sequentially collected in four wells with 0.1 M PBS at 4°C. Before the incubation with specific antibodies, slices were pre-treated with a solution containing 1% of hydrogen peroxide and 10% methanol diluted in 0.1 M PBS for 30 min at 4°C. After 3 10-min washes in 0.1 M PBS, the slices were pre-incubated in 2% normal donkey serum (S30-10ML, Millipore Temceula, CA, USA) for 1 h. Slices were then incubated on a shaker for 48 h at 4°C with the following primary antibodies: *mouse anti-rat blood-brain barrier monoclonal antibody* (*SMI 71*, 1:2,000; Covance, CA, USA); *rabbit anti-angiotensin type 1 receptor (AT1 (306):sc-579*, 1:50, Santa Cruz Biotechnology, CA, USA) plus *mouse anti-neuronal nuclei (NeuN)* (1:1,000; Millipore, Darmstadt, German); or *rabbit anti-mouse IBA-1* (1:1,000; Wako, Osaka, Japan). Primary antibodies were diluted in 0.1 M PBS containing 0.1% Triton X-100. Tissue slices were then incubated for 1 h at room temperature with secondary antibodies (Jackson Immunoresearch Laboratories Inc., MD, USA): *Alexa Fluor 488-conjugated AffiniPure donkey anti-mouse IgG* (H+L), 1:500 dilution, *Alexa Fluor 594-conjugated AffiniPure donkey anti-*rabbit *IgG* (H+L), 1:500 dilution or *Alexa Fluor 488-conjugated AffiniPure donkey anti-rabbit IgG* (H+L), 1:500 dilution. Tissue slices in the 4th well were used for negative controls (absence of primary or secondary antibody). After 3 other 10-min washes with 0.1 M PBS, the slices were mounted in gelatinized slides (*Slowfade*® *Gold Antifade*) and stored in dark boxes at 4°C. Images from all experimental groups were digitized with identical acquisition settings and analyzed (Leica DMLB) by an investigator blind to group allocation. A threshold paradigm was used for normalization and quantification of the immunofluorescence signal, as previously described (Higa-Taniguchi et al., 2007). Briefly an averaged background staining was obtained from each image and only signal intensities 1.5-times higher than the calculated background fluorescence were considered for the analysis. Immunofluorescence signals (integrated density) were measured within the ventromedial, dorsal cap, and posterior autonomic subnuclei of the PVN (AOIs of 28,884, 5,135, 46,405 μm^2^, respectively), NTS (AOI of 48,378 μm^2^), and RVLM (AOI of 42,168 μm^2^) and expressed in arbitrary units/μm^2^. Quantifications were performed in both left and right side of the nuclei; values from sections were averaged to give a mean value for the nucleus for each rat in each condition.

### Statistical analysis

Data are presented as mean ± SEM. MAP and BBB leakage changes in WKY and SHR groups during life span and treadmill performance during S and T conditions were analyzed by two- and three-way ANOVA with repeated measurements (time), respectively. Training effects in SHR-T and WKY-T during the 8-weeks protocol were analyzed by the factorial ANOVA. The comparison of functional data and BBB leakage area between groups (SHR-S, SHR-T, WKY-S, and WKY-T) at different time points (weeks 0 and 8) was made by two-way ANOVA. Comparison of BBB leakage in rats infused with saline or Ang II was analyzed by one-way ANOVA. Unpaired *t*-test as used to compare hypoglossus BBB leakage area between SHR and WKY groups. Fisher was used as the *post-hoc* test. Correlation analyses were performed using Pearson's statistics. Bonferroni correction was used to ratify the significance level for the different correlations made. All analyses were performed using the STATISTICA 12.0 (Vince Stat Software Inc). Differences were considered significant at *P* < 0.05.

## Results

### Blood pressure and BBB permeability changes during development in SHR and WKY

**Figure 1:**
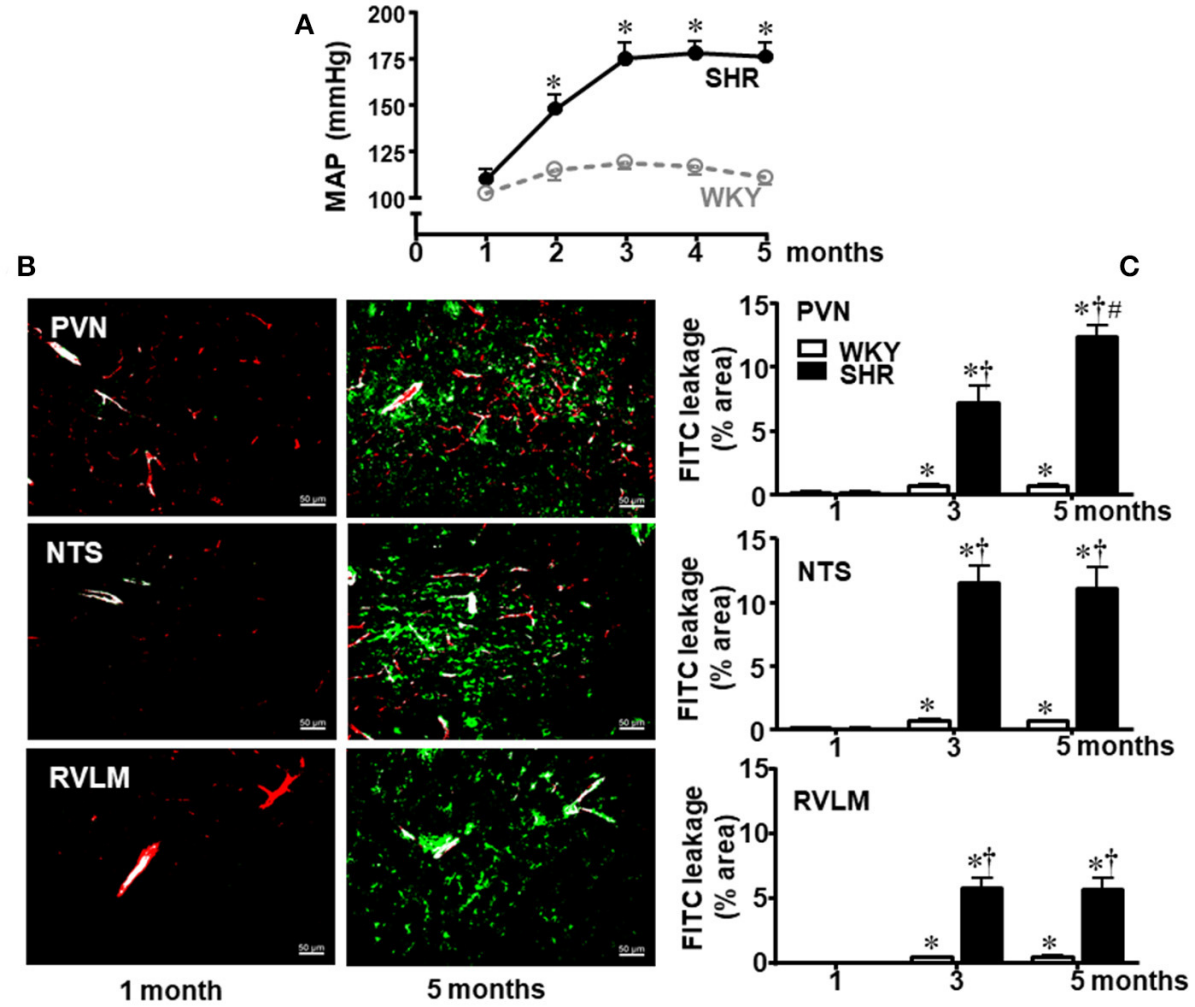
Temporal changes of blood-brain barrier permeability within autonomic areas during 5 months' life span in sedentary spontaneously hypertensive (SHR) and normotensive rats (WKY). **(A)** Resting mean arterial pressure (MAP) values in SHR and WKY during 5 months' life span. *n* = 8 rats/subgroup. Significances (^*^P < 0.05) vs. WKY. **(B)** Images show the capillary profile (red, Rhodamine) and FITC extravasation (green) into the brain parenchyma in the paraventricular nucleus of the hypothalamus (PVN), nucleus of the tract solitary (NTS), and rostral ventrolateral medulla (RVLM) in SHR aged 1 and 5 months. **(C)** Graphs compare FTIC leakage changes within the three autonomic areas of SHR and WKY groups from the 1st up to the 5th month. Values are the means of 8–12 slices, 4 rats in each subgroup. Significances (*P* < 0.05) ^*^vs. 1st month; ^†^vs. age-matched WKY; ^#^vs. SHR aged 3 months.

Our first approach was to analyze time-course changes on BBB permeability during life span since the pre-hypertensive phase. Resting mean AP (MAP) was measured in conscious sedentary SHR and WKY aged 1, 2, 3, 4, and 5 months (Figure 1A) and the integrity of BBB in autonomic brain areas was evaluated at the 1st, 3rd, and 5th month (Figures 1B,C). There was no significant BBB leakage in WKY and SHR aged 1 month. In the sedentary WKY, 5-months life span was accompanied by very small although significant increases in BBB leakage (0.7 ± 0.1, 0.7 ± 0.1, and 0.5 ± 0.1% in the PVN, NTS, and RVLM, respectively, at the 5th vs. 1st month, Figure 1C). In contrast, the establishment of hypertension was accompanied by marked increases in FITC extravasation within the brain parenchyma (*P* < 0.001 for group, age and interaction in the three areas analyzed): in SHR aged 3 months, dye leakage was 7.2 ± 1.3, 11.5 ± 1.3, and 5.8 ± 0.8% within the PVN, NTS, and RVLM, respectively (Figure 1C), corresponding to increases over 100 times, when compared to age-matched WKY. From the early to a late phase of hypertension, leakage area was unaltered in the NTS and RVML, but it increased in the PVN (12.4 ± 0.9% at the 5th month). Interestingly, hypertension-induced BBB disruption was not observed in the hypoglossus nucleus, since FITC extravasation was small and similar in both sedentary strains aged 3 months (WKY = 0.23 ± 0.05%; SHR = 0.24 ± 0.06%).

### Temporal effects induced by training on hemodynamic parameters and their variabilities in adult SHR and WKY

SHR and WKY aged 3 months were submitted to training (T) or sedentary (S) protocols. Besides the better treadmill performance exhibited by the SHR since the beginning of protocols, both T groups showed proportional increases in attained velocities and similar performance gain at the end of protocols (+73% for SHR-T and WKY-T vs. respective values at week 0, *P* = 0.003 for time, *P* < 0.001 for group, condition and interaction effects, Table 1). The performance gain was not changed in WKY-S, but significantly decreased in SHR-S (−40%, Table 1). Both groups exhibited similar body weight at week 0. Except for the SHR-S that exhibited a slight higher value, body weight gain during T and S protocols was similar in the other groups (Table 1).

**Table 1:** Treadmill performance and body weight of WKY and SHR groups during training (T) or sedentary (S) protocols.

	**WKY-*S***	**WKY-T**	**SHR-S**	**SHR-T**
**TREADMILL PERFORMANCE DURING S AND T PROTOCOLS**				
week 0 (km/h)	1.1 ± 0.1		1.5 ± 0.2^*^	
	(*n* = 42)		(*n* = 74)	
week 4 (km/h)	1.2 ± 0.1 (*n* = 8)	1.7 ± 0.1^#^^†^ (*n* = 16)	1.1 ± 0.2^#^ (*n* = 12)	2.3 ± 0.1^#^^†^^*^ (*n* = 34)
week 8 (km/h)	1.0 ± 0.0 (*n* = 8)	1.9 ± 0.2^#^^†^ (*n* = 8)	0.9 ± 0.1^#^ (*n* = 12)	2.6 ± 0.2^#^^†^^*^ (*n* = 14)
gain (% change)	−8	+73	−40	+73
**BODY WEIGHT DURING S AND T PROTOCOLS**				
week 0 (g)	265 ± 5		261 ± 2	
	(*n* = 42)		(*n* = 74)	
week 4 (g)	298 ± 9^#^ (*n* = 8)	303 ± 9^#^ (*n* = 16)	303 ± 5^#^ (*n* = 12)	294 ± 4^#^ (*n* = 34)
week 8 (g)	304 ± 8^#^ (*n* = 8)	311 ± 9^#^ (*n* = 8)	329 ± 5^#^ (*n* = 12)	305 ± 5^#^^†^ (*n* = 14)
BW gain (g)	+42 ± 7	+45 ± 6	+65 ± 5^*^	+43 ± 4^†^

Values are means±SEMs. Gain represents the difference between experimental weeks 8 and 0. The number of rats is indicated in parenthesis. Significances (P < 0.05) are

* *vs. WKY*;

# *vs. week 0*;

† *vs. respective S group*.

SHR had already attained the established phase of hypertension at the beginning of T and S protocols (average increases of 23 and 11% in resting MAP and HR, vs. respective values in WKY, Table 2). Training affected differentially resting values of MAP (*P* < 0.001 for group and condition; time, *P* = 0.045; interaction, *P* = 0.269) and HR (*P* < 0.001 for group, condition and time; *P* = 0.278 for interaction). SHR-T exhibited significant MAP fall (−13%) and resting bradycardia (−13%) from T_4_ up to T_8_, while age-matched WKY-T only showed resting bradycardia (−12% from T_1_ on, Table 2) when compared to respective sedentary controls. Although AP and HR values were not changed in S groups, SHR-S_8_ showed a 2.2-fold increase in SAP variability when compared to SHR-S_0_ (*P* < 0.001 for group and condition; *P* = 0.122 for time; *P* = 0.006 for interaction, Table 3). At the end of protocols, SHR-S_8_ vs. WKY-S_8_ exhibited higher sympathetic vasomotor modulation (LF-SAP increased by 5.1-fold, Table 3). These effects were completely abrogated by exercise training, with a significant reduction of LF-SAP (−45%) since T_2_ (*P* < 0.001 for group and condition; *P* = 0.035 for time; *P* = 0.241 for interaction). Pulse interval (PI) variability was almost similar in sedentary groups, unaffected during the 8-weeks sedentary protocol, but augmented in both trained strains (*P* = 0.115 for group; *P* = 0.017 for condition; *P* < 0.001 for time; *P* = 0.505 for interaction): PI variability was significantly increased in SHR-T_4_ and SHR-T_8_ (2.8- and 4.4-fold increases, respectively) and in WKY-T_4_ (2.5-fold, Table 3). Although similar increases in PI variability (~2.0-fold change) were observed in WKY at the 1st, 2nd, and 8th weeks of training, the large individual variabilities precluded significant changes. Interestingly, training-induced increase on PI variability was caused by different mechanisms in each strain (Table 3): an early and maintained (T_2_ and T_8_) elevation of HF-PI in the SHR and a late (T_8_) reduction of LF-PI in the WKY. Spontaneous baroreflex sensitivity (as indicated by αLF and αHF indexes in Table 3), was depressed in the SHRs vs. WKYs and improved by exercise training in both groups (*P* < 0.001 for group and condition; *P* = 0.045 for time; *P* = 0.723 for interaction effect). These training-induced changes in autonomic modulation significantly reduced the LF/HF ratio in both trained groups.

**Table 2:** Time course changes of resting arterial pressure (AP) and heart rate (HR) in sedentary (S) and trained (T) WKY and SHR groups during the 8 experimental weeks.

**Groups**	**SAP (mmHg)**	**DAP (mmHg)**	**MAP (mmHg)**	**HR (b/min)**
**WKY**				
S_0_ = T_0_	146 ± 4	113 ± 3	128 ± 2	363 ± 18
T_1_	148 ± 5	116 ± 4	128 ± 3	320 ± 11^#^
T_2_	146 ± 6	112 ± 5	126 ± 5	312 ± 13^#^
T_4_	149 ± 8	113 ± 5	127 ± 8	303 ± 16^#^
T_8_	147 ± 6	114 ± 4	127 ± 5	321 ± 10^#^^†^
S_8_	145 ± 4	110 ± 3	123 ± 3	347 ± 15
**SHR**				
S_0_ = T_0_	198 ± 9^*^	156 ± 6^*^	175 ± 7^*^	404 ± 11^*^
T_1_	186 ± 7^*^	145 ± 6^*^	161 ± 6^*^	383 ± 12^*^
T_2_	179 ± 10^*^	141 ± 9^*^	157 ± 10^*^	366 ± 7^*^
T_4_	173 ± 7^*^	138 ± 8^*^	153 ± 7^^#^^^*^	351 ± 11^*^^^#^^
T_8_	170 ± 6^#^^†^^*^	137 ± 5^#^^†^^*^	153 ± 6^#^^†^^*^	355 ± 5^*^^†^#^^
S_8_	189 ± 8^*^	148 ± 6^*^	176 ± 8^*^	392 ± 13^*^

Values are means±SEM; SAP, systolic AP; DAP, diastolic AP; MAP, mean AP. Hemodynamic measurements were made in 4-5 WKY/subgroup and 8–10 SHR/subgroup. Significances (P < 0.05) are

* *vs. week-matched WKY*;

# *vs. respective week 0*;

† *vs. respective S_8_*.

**Table 3:** Changes in systolic arterial pressure (SAP) and pulse interval (PI) variabilities and respective spectral components induced by training (T) or sedentary (S) protocols in adult SHR and WKY groups.

**Groups/ weeks**	**SAP variability (mmHg^2^)**	**LF-SAP (mmHg^2^)**	**PI variability (ms^2^)**	**LF-PI (ms^2^)**	**HF-PI (ms^2^)**	**LF/HF ratio**	**α LF (ms^2^/mmHg^2^)**	**α HF (ms^2^/mmHg^2^)**
**WKY**								
S_0_ = T_0_	40.39 ± 2.81	4.69 ± 0.93	38.87 ± 12.48	1.90 ± 0.21	9.69 ± 0.78	0.20 ± 0.02	0.65 ± 0.09	1.59 ± 0.55
T_1_	33.31 ± 3.68	4.22 ± 1.32	79.72 ± 16.45	2.66 ± 0.71	11.55 ± 2.46	0.22 ± 0.02	1.16 ± 0.28	2.95 ± 0.67
T_2_	29.84 ± 5.18	3.47 ± 0.45	77.16 ± 19.35	2.82 ± 1.01	10.56 ± 2.19	0.21 ± 0.04	1.05 ± 0.10^#^	3.03 ± 0.56^#^
T_4_	31.52 ± 3.20	3.09 ± 0.49	80.18 ± 6.90^#^	2.37 ± 0.64	11.18 ± 1.26	0.21 ± 0.05	1.06 ± 0.17^#^	2.93 ± 0.77
T_8_	30.95 ± 2.57	2.70 ± 0.85	92.22 ± 19.94	1.43 ± 0.19^†^^#^	9.15 ± 1.03	0.16 ± 0.02^†^	1.09 ± 0.14^#^^†^	3.27 ± 0.41^#^^†^
S_8_	43.73 ± 9.22	4.70 ± 0.48	51.50 ± 7.54	2.99 ± 0.52	10.29 ± 1.93	0.27 ± 0.06	0.67 ± 0.07	2.08 ± 0.26
**SHR**								
S_0_ = T_0_	59.32 ± 7.47^*^	17.54 ± 3.29^*^	23.89 ± 3.69	2.43 ± 0.36	8.61 ± 0.88	0.24 ± 0.02	0.24 ± 0.03^*^	1.01 ± 0.20
T_1_	56.42 ± 8.23^*^	11.11 ± 2.24^*^	49.04 ± 3.07	2.67 ± 0.63	8.72 ± 1.24	0.28 ± 0.04	0.41 ± 0.09^*^	1.23 ± 0.16^*^
T_2_	49.51 ± 8.81^*^	10.27 ± 1.75^*^^#^	50.83 ± 13.29	1.83 ± 0.44	14.50 ± 1.66^#^	0.15 ± 0.02^#^	0.41 ± 0.08^*^	1.27 ± 0.21^*^
T_4_	48.34 ± 4.62^*^	9.03 ± 1.23^*^^#^	67.66 ± 8.27^#^	1.84 ± 0.41	12.32 ± 2.87	0.17 ± 0.03^#^	0.57 ± 0.07^*^^#^	1.57 ± 0.16
T_8_	45.62 ± 4.97^*^^†^	9.62 ± 1.38^*^^#^^†^	106.12 ± 13.27^#^^†^	1.44 ± 0.28	16.30 ± 3.69^*^^#^^†^	0.16 ± 0.01^#^^†^	0.55 ± 0.07^*^^#^^†^	2.15 ± 0.54^#^^†^
S_8_	128.14 ± 20.29^*^^#^	24.15 ± 5.96^*^	38.54 ± 6.80	2.06 ± 0.31	7.79 ± 0.68	0.24 ± 0.03	0.29 ± 0.04^*^	0.88 ± 0.13^*^

Values are means±SEMs. LF, low-frequency component; HF, high-frequency component. αLF and αHF represent the spontaneous baroreflex sensitivity calculated with the LF or HF components of PI and corresponding SAP components. Measurements were made in 4–5 WKY/subgroup and 8-10 SHR/subgroup. Significances (P < 0.05) are

* *vs. week-matched WKY*;

# *vs. respective week 0*;

† *vs. respective S_8_*.

### Temporal effects induced by training on BBB permeability in adult SHR and WKY

**Figure 2:**
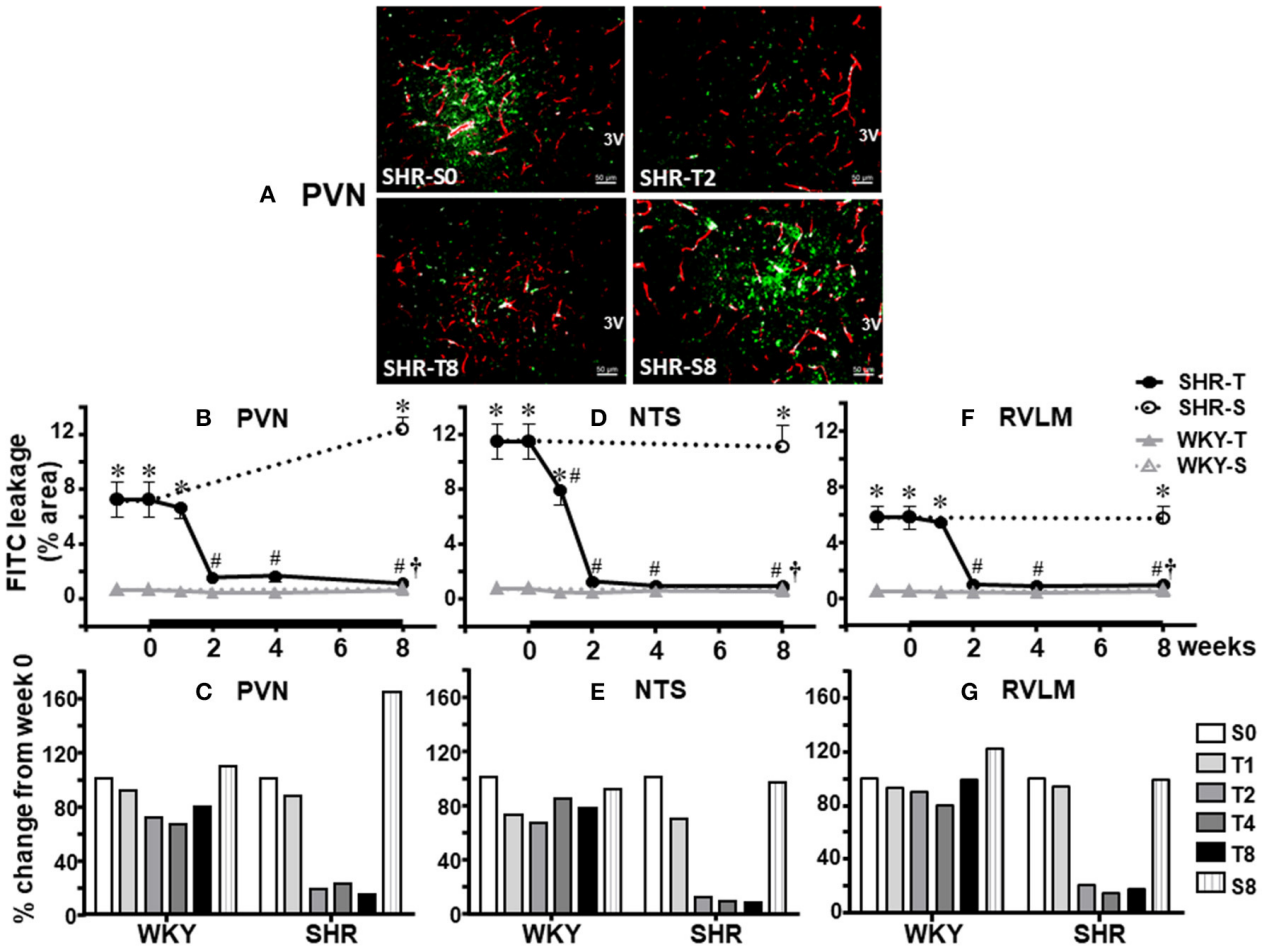
Temporal changes of blood-brain barrier permeability induced by aerobic training in autonomic areas of spontaneously hypertensive (SHR) and normotensive rats (WKY) aged 3 months. **(A)** Representative image showing the capillary profile (red) and the effects of 2 and 8 weeks of exercise training on FITC extravasation (green) within the brain parenchyma of the paraventricular nucleus of the hypothalamus (PVN). For comparison images of the sedentary (S) SHR are shown at weeks 0 and 8. Graphs depict training-induced changes on absolute leakage area **(B,D,F)** and percent changes from values at week 0 **(C,E,G)** observed within the PVN **(B,C)**, nucleus of the solitary tract (NTS, **D,E**) and the rostral ventrolateral medulla (RVLM, **F,G**) of the SHR and WKY groups. Values are the means of 8–12 slices, 4 rats in each subgroup. Significances (*P* < 0.05) are ^*^vs. WKY; ^#^vs. week 0; ^†^vs. respective S groups.

As documented in Figure 1, SHR aged 3 months exhibited marked FITC leakage within the brain parenchyma of the three autonomic areas. Photomicrographs and quantitative PVN data obtained during S and T protocols show that (*P* < 0.001 for group, condition, time, and interaction): (i) the leakage area was significantly increased in SHR kept sedentary for 8 weeks (+ 72% from S_0_ to S_8_, Figures 2A,B); (ii) training caused a prompt and marked improvement on BBB integrity in the PVN from T_2_ on (−79%, Figure 2B); (iii) BBB leakage area was reduced by 91% in SHR-T_8_ when compared to SHR-S_8_ (Figure 2C); (iv) WKY-T also showed a small improvement on BBB integrity (FITC leakage was reduced by 28%, Figure 2C).

Except for the maintained leakage during the 8-weeks S protocol, similar patterns were observed in the NTS and RVLM of the SHR group (illustrative images were not shown). Quantitative data (*P* < 0.001 for group and condition; *P* = 0.501 for time; *P* = 0.023 for interaction) confirmed that 2 weeks of training significantly reduced both NTS (−89%, *P* < 0.05, Figure 2D) and RVLM leakage area (−83%, *P* < 0.05, Figure 2F), values that were maintained up to the end of protocols. Exercise training also caused small reduction on FITC leakage within the NTS and RVLM of the WKY group (−24 and −11%, Figures 2E,G, respectively).

### Training-induced improvement of BBB integrity correlates with the amelioration of autonomic function

**Figure 3:**
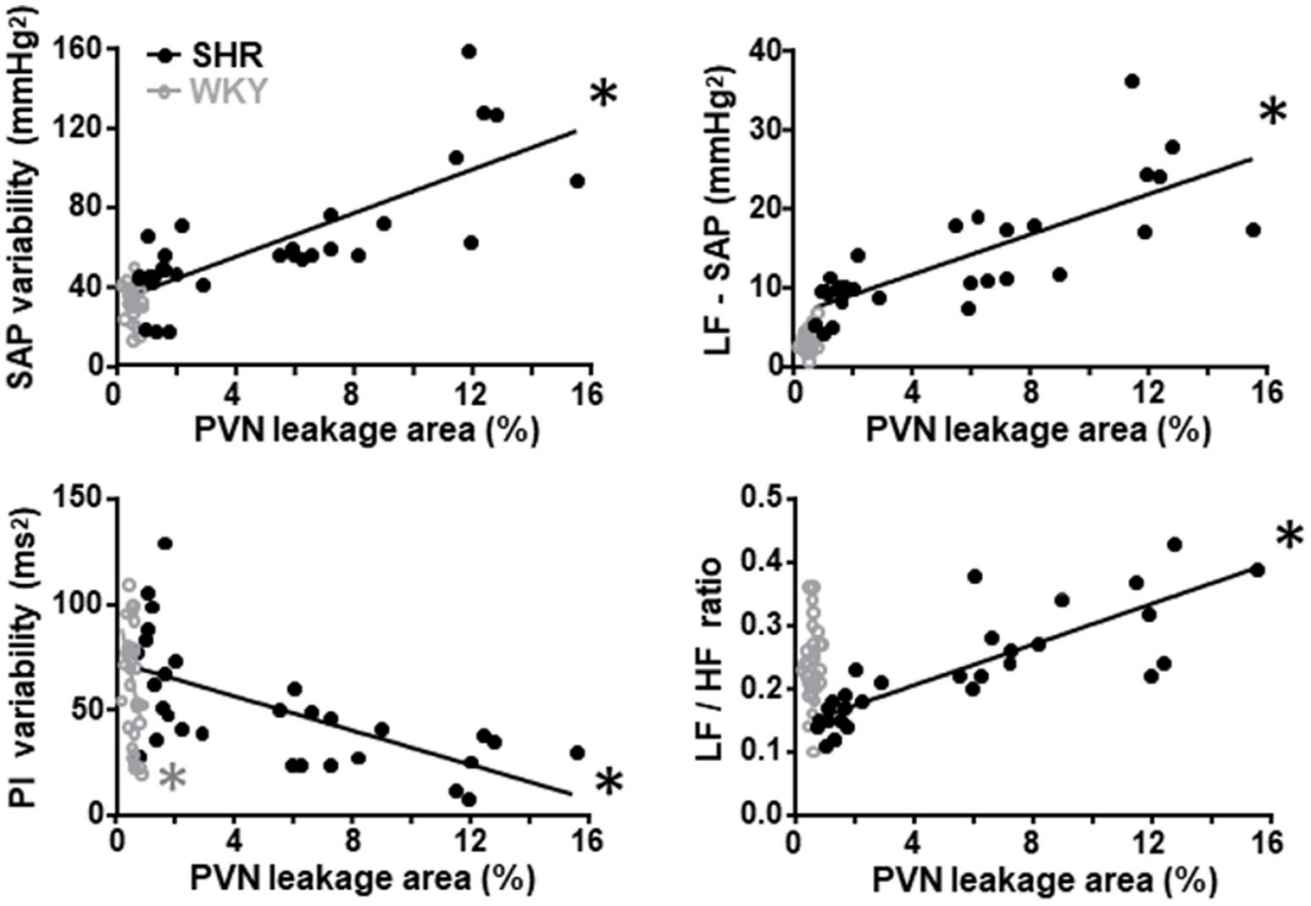
Correlations between PVN blood-brain barrier permeability and autonomic parameters in sedentary and trained spontaneously hypertensive (SHR) and normotensive rats (WKY). In the SHR group, training-induced reduction in the leakage area was positively correlated with reduced systolic arterial pressure (SAP) variability, with its low frequency component (LF) and with decreased LF/HF ratio to the heart. Reduced leakage area was negatively correlated with increased pulse interval (PI) variability. Correlation coefficients and *P*-values for regression equations are presented in Table 4. ^*^denotes a significant correlation.

Since the preservation of BBB integrity in autonomic nuclei of trained rats and the improvement and/or correction of autonomic modulation exhibit similar time course changes, we evaluate whether these parameters are correlated. Training-induced reduction of the PVN leakage area was positively correlated with the decrease of both LF-SAP (an index of sympathetic vasomotor outflow) and SAP variability only in the SHR group (Figure 3; correlation coefficients and P values are presented in Table 4). Reduced PVN leakage area was positively correlated with decreased LF/HF ratio to the heart and negatively correlated with spontaneous baroreflex sensitivity in the SHR group (Figure 3, Table 4). On the other hand, reduction of PVN leakage area was negatively correlated with increased PI variability in both SHR and WKY groups. Similar correlations between improvement of autonomic functions and improvement of BBB integrity were observed for the NTS and RVLM in the hypertensive strain (Table 4).

**Table 4:** Correlation coefficients (*r*) and *P*-values of regression equations correlating training-induced changes on autonomic control with training-induced changes on BBB leakage within the PVN, NTS, and RVLM of WKY and SHR groups.

	**Groups**	
	**WKY**	**SHR**
**PVN**		
BBB leakage x SAP variability	*r* = −0.140 *P* = 0.461	*r* = 0.778 *P* < 0.001^#^
BBB leakage x LF-SAP	*r* = 0.337 *P* = 0.069	*r* = 0.779 *P* < 0.001^#^
BBB leakage x PI variability	*r* = −0.421 *P* = 0.021	*r* = −0.632 *P* < 0.001^#^
BBB leakage x LF/HF ratio	*r* = 0.075 *P* = 0.696	*r* = 0.827 *P* < 0.001^#^
BBB leakage x α LF	*r* = 0.040 *P* = 0.832	*r* = −0.587 *P* < 0.001^#^
BBB leakage x α HF	*r* = −0.312 *P* = 0.094	*r* = −0.417 *P* = 0.022
**NTS**		
BBB leakage x SAP variability	*r* = 0.215 *P* = 0.255	*r* = 0.744 *P* < 0.001^#^
BBB leakage x LF-SAP	*r* = 0.307 *P* = 0.099	*r* = 0.732 *P* < 0.001^#^
BBB leakage x PI variability	*r* = −0.130 *P* = 0.495	*r* = −0.686 *P* < 0.001^#^
BBB leakage x LF/HF ratio	*r* = −0.116 *P* = 0.542	*r* = 0.686 *P* < 0.001^#^
BBB leakage x α LF	*r* = −0.151 *P* = 0.427	*r* = −0.656 *P* < 0.001^#^
BBB leakage x α HF	*r* = −0.393 *P* = 0.032	*r* = −0.466 *P* = 0.010
**RVLM**		
BBB leakage x SAP variability	*r* = 0.187 *P* = 0.323	*r* = 0.605 *P* < 0.001^#^
BBB leakage x LF-SAP	*r* = 0.298 *P* = 0.110	*r* = 0.731 *P* < 0.001^#^
BBB leakage x PI variability	*r* = 0.065 *P* = 0.733	*r* = −0.616 *P* < 0.001^#^
BBB leakage x LF/HF ratio	*r* = 0.061 *P* = 0.749	*r* = 0.723 *P* < 0.001^#^
BBB leakage x α LF	*r* = 0.354 *P* = 0.055	*r* = −0.525 *P* = 0.003
BBB leakage x α HF	*r* = −0.099 *P* = 0.601	*r* = −0.404 *P* = 0.027

*SAP, Systolic arterial pressure; PI, pulse interval; LF, low-frequency component; HF, high-frequency component; αLF and αHF, spontaneous baroreflex sensitivity estimated by LF and HF spectral components respectively. Correlations include 24–30 values of sedentary and trained rats (n = 4 rats/subgroup) from all time points*.

# *denotes a significant correlation after Bonferroni correction of the significant level (α_c_ = 0.0028) for the 18 analysis made*.

### Combined effects of training and *icv* Ang II infusion on BBB permeability in adult SHR

Because main changes on BBB permeability were observed in the hypertensive strain and knowing that high Ang II availability increases (Biancardi et al., 2014) while exercise training decreases BBB leakage (present set of data), next we analyzed in the SHR the combined effects of both. According to our previous studies (Masson et al., 2014, 2015; Chaar et al., 2015) and the present observations showing that training-induced autonomic adaptations as well as improvement of BBB integrity in the SHR occurred after 2 weeks of exercise training, we extended this protocol for 14 experimental days. Notice that 14 daily consecutive sessions of exercise correspond to the volume of training attained between experimental weeks 2 and 3 of the previous protocol in which the rats trained 5 days/week.

**Figure 4:**
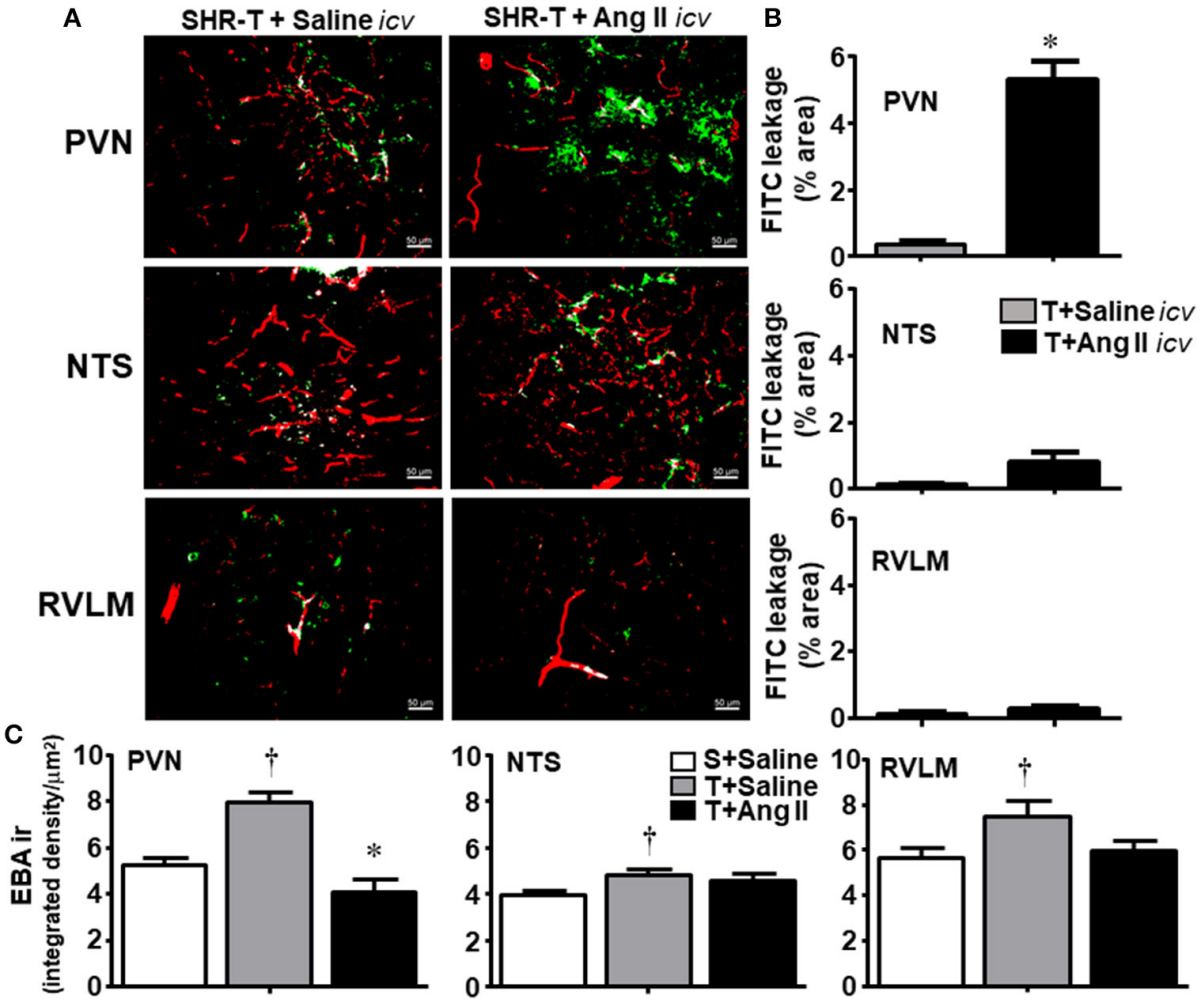
Combined effect of aerobic training (T) and angiotensin II (Ang II) infusion on blood-brain barrier permeability in autonomic areas of spontaneously hypertensive rats (SHR) aged 3 months. **(A)** Representative images showing capillary profile (red) and FITC extravasation (green) within the brain parenchyma of the paraventricular nucleus of the hypothalamus (PVN), nucleus of the solitary tract (NTS) and rostral ventrolateral medulla (RVLM) in SHR-T infused with saline or Ang II *icv*. **(B)**. Quantification of the leakage area in the three autonomic nuclei of SHR-T infused with saline or Ang II *icv*. Measurements were made in 7–9 slices, 3–4 rats/group. Significance (*P* < 0.05) ^*^vs. saline. **(C)** Effects of T and T associated with Ang II *icv* infusion on the expression of endothelial barrier antigen (EBA ir) within the PVN, NTS, and RVLM. EBA ir in the sedentary (S) SHR infused with saline is shown for comparison. Significances (*P* < 0.05) are ^†^vs. S+saline; ^*^vs. T+ saline.

SHR-T simultaneously infused with *icv* sterile saline during 14-days showed very small FITC leakage into the brain parenchyma of the PVN, NTS, and RVLM (Figures 4A,B), a response similar to that observed previously. In contrast, SHR-T infused with Ang II *icv* for 14 days exhibited significant BBB leakage within the PVN (5.3 ± 0.6%, Figures 4A,B). Interestingly, 14-days Ang II infusion caused small increases but did not significantly change FITC leakage area within the NTS and RVLM of the SHR-T. To confirm the effects of Ang II and exercise training on the lesion and/or integrity of the BBB, next we analyzed the expression of endothelial barrier antigen (EBA), a luminal protein expressed only in cerebral microvessels with intact BBB (Lin and Ginsberg, 2000; Norsted et al., 2008). When compared to SHR-S, EBA staining was significantly elevated in the three autonomic areas of the SHR-T, with a larger increase in the PVN (Figure 4C). Accordingly, simultaneous Ang II treatment blocked the effect of training.

**Figure 5:**
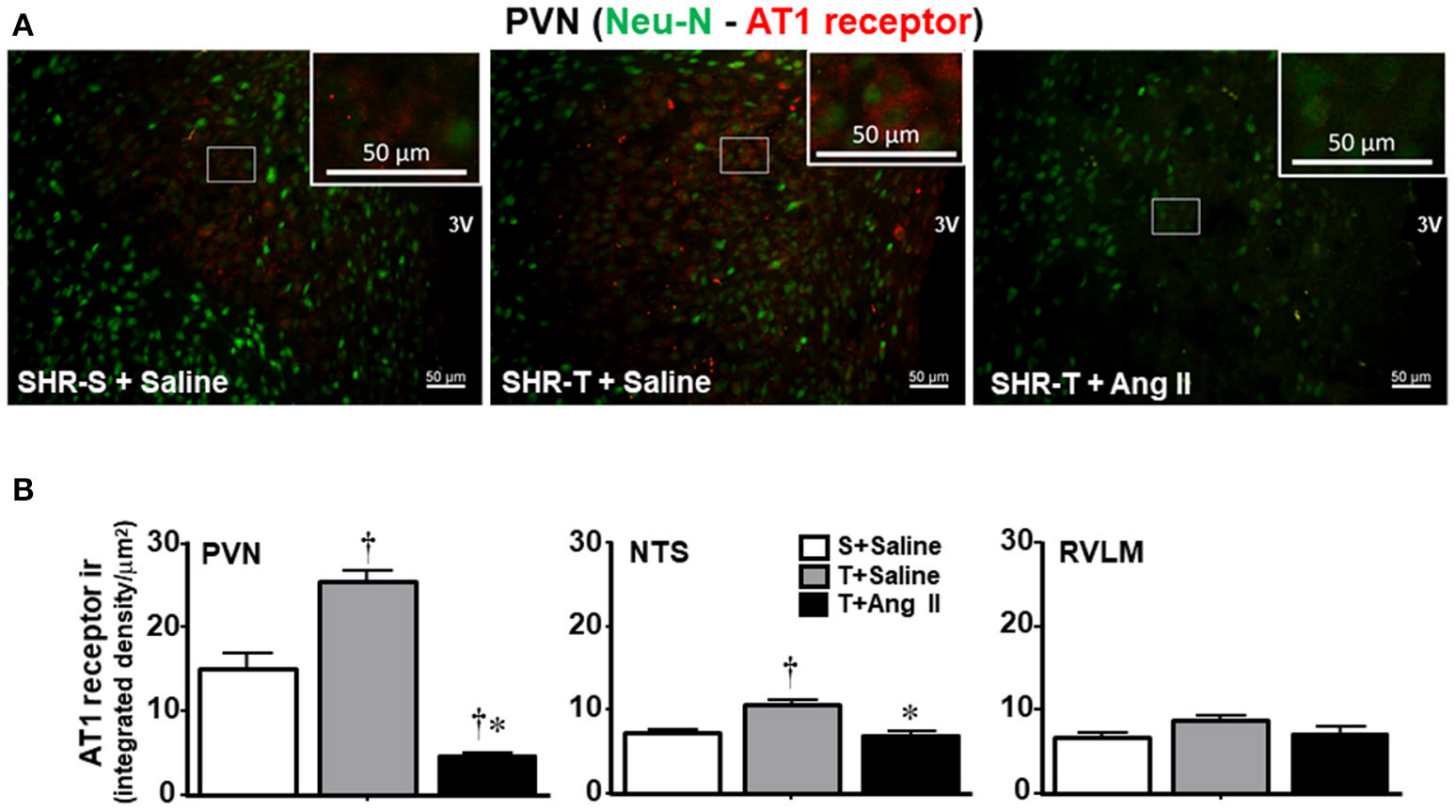
Combined effect of aerobic training (T) and angiotensin II (Ang II) infusion on AT1 receptor expression in autonomic nuclei of spontaneously hypertensive rats (SHR) aged 3 months. **(A)** Representative images comparing AT1 receptor expression (red) within the paraventricular nucleus of the hypothalamus (PVN) in SHR-T infused with saline or angiotensin II (Ang II). Neurons are identified by Neu-N staining (green). Insets are high magnification of the white rectangle depicting representative neurons that show increased receptors' expression in SHR-T+Saline and decreased in SHR-T+Ang II when compared to SHR-S+saline. **(B)** Quantification of AT1 receptor density within the PVN, nucleus of the solitary tract (NTS) and rostral ventrolateral medulla (RVLM) in SHR-T infused with saline or Ang II *icv*. AT1 receptor expression in the sedentary (S) SHR infused with saline is also shown for comparison. Measurements were made in 7–9 slices, 3–4 rats/group. Significances (*P* < 0.05) are ^†^vs. S+saline; ^*^vs. T+ saline.

**Figure 6:**
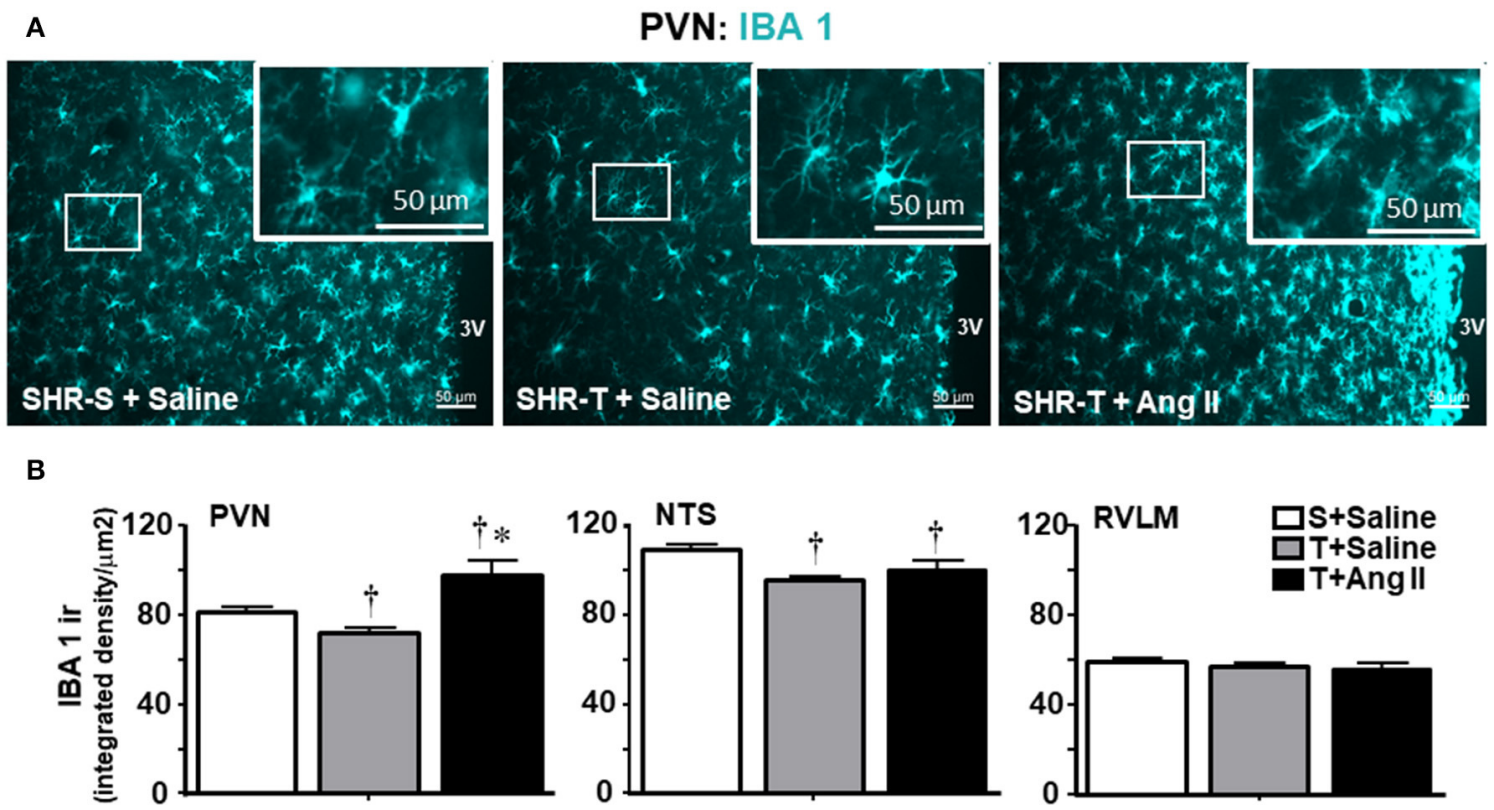
Combined effect of aerobic training (T) and angiotensin II (Ang II) infusion on microglia expression (IBA1 ir) in autonomic nuclei of spontaneously hypertensive rats (SHR) aged 3 months. **(A)** Representative images comparing IBA1 expression (blue) within the paraventricular nucleus of the hypothalamus (PVN) in SHR-T infused with saline or angiotensin II (Ang II). Insets depict high magnification of areas delimited by the white rectangle and show both the maintenance of inactivated microglia in SHR-T+Saline and activated pattern in SHR-T+Ang II. **(B)** Quantification of IBA1 density in the PVN, nucleus of tract solitary (NTS) and rostral ventrolateral medulla (RVLM) in SHR-T infused with saline or Ang II *icv*. IBA1 expression in the sedentary (S) SHR infused with saline is also shown for comparison. Measurements were made in 7–9 slices, 3–4 rats/group. Significances (*P* < 0.05) are ^†^vs. S+saline; ^*^vs. T+ saline.

Knowing that BBB disruption facilitates the access of circulating Ang II to neurons and microglial cells (Biancardi et al., 2014), next we analyzed the effects of training and Ang II treatment on the expression of AT_1_ receptors in neurons and on microglia activation within the PVN, NTS, and RVLM. Unexpectedly, training was accompanied by an upregulation of AT_1_ receptors in PVN neurons, an effect that was blocked by simultaneous Ang II *icv*. infusion (Figures 5A,B). Again, training and Ang II effects were more prominent within the PVN when compared to NTS and RVLM (quantitative data in Figure 5B; illustrative images were not shown). Photomicrographs of microglia expression in the PVN of SHR-T treated with saline (Figure 6A) show that training markedly reduced its density and kept microglial cells in an inactive state (long and ramified processes), while this response was completely blocked in SHR-T treated with Ang II that exhibited increased microglia expression and activated microglial cells (rhomboid shape, with short and thicker processes—insets in Figure 6A). Quantitative analysis ratified the attenuation of microglia activation within the PVN and NTS of the SHR-T (reduced IBA-1 expression, Figure 6B), without significant changes in the RVLM (illustrative images of IBA 1 staining in the NTS and RVLM were not shown). The blockade of training effect within the PVN by simultaneous infusion of Ang II confirmed this effect was mediated by reduced local availability of the neurohormone in the SHR-T and that Ang II effects were faster within this autonomic area.

## Discussion

The present set of data confirm previous observations of hypertension-induced BBB disruption in brain areas modulating the autonomic control of the circulation (Biancardi et al., 2014; Biancardi and Stern, 2016) showing in addition a new finding—the complete blockade of this deleterious effect by the exercise training, even in the presence of hypertension. There are several original observations: (i) BBB disruption is absent in the PVN, NTS, and RVLM of the SHR at the pre-hypertensive phase, but fully manifested in the established phase of hypertension; (ii) within the PVN there is a progressive increase in BBB dysfunction during the maintained phase of hypertension, an effect not observed in other autonomic nuclei; (iii) exercise training rapidly reverses dye leakage, increases the expression of endothelial barrier antigen and almost normalizes PVN, NTS, and RVLM BBB permeability, an effect strongly correlated with reduced sympathetic vasomotor activity, decreased pressure variability, and increased heart rate variability observed in the trained SHR; (iv) training-induced reduction of brain Ang II availability (with decreased microglia activation) contributes to the improvement of BBB integrity in the trained SHR since simultaneous Ang II infusion blocks these responses; (v) Ang II blockade of training effects on BBB permeability is a prompt response in the PVN with minor effects in other autonomic areas after 14-days stimulation; (vi) in the WKY group repetitive exercise also induces a mild reduction of BBB permeability within the PVN and NTS, effects significantly correlated with the establishment of resting bradycardia in the normotensive strain.

The BBB, a complex multicellular structure acting as a selective barrier between the systemic circulation and the central nervous system, restricts the entry of substances into the brain by the transcellular (mediated by specific transporters and receptor proteins across the endothelial cell) and the paracellular transport (high electrical resistance tight junctions). Several techniques (dyes and/or radioactive substances' permeability, transendothelial electrical resistance, immunohistochemistry, RT-PCR, western blotting, electron microscopy) have been used to quantify BBB integrity/disruption and the expression of BBB constituents. Although BBB leakage induced by either hypertension, stroke, hyperosmolar stimulus, and aging has been reported in brain areas related to cognitive impairment since the 80's (Mueller and Heistad, 1980; Tamaki et al., 1984; Amenta et al., 2003; Ueno et al., 2004; Al-Sarraf et al., 2007; Zhang et al., 2010; Capone et al., 2011; Faraco and Iadecola, 2013; Iadecola, 2015), information on the conditioning mechanism(s) are scarce. Regarding BBB leakage in hypertension, there are conflicting evidence since studies reported loss of tight junction proteins within the cortex and hippocampus (Pelisch et al., 2011; Mohammadi and Dehghani, 2014; Zhang et al., 2015) as well as no evidence of ultrastructural tight junctions' disruption (Ueno et al., 2004). Absence of tight junction breakdown was also reported in rats submitted to focal cerebral ischemia (Krueger et al., 2013). Recent studies have shown that hypertension is accompanied by increased BBB permeability within the PVN, NTS, and RVLM, important autonomic areas controlling sympathetic and parasympathetic outflow to heart and blood vessels (Yao and May, 2013; Biancardi et al., 2014; Biancardi and Stern, 2016). Our data showing increased FITC leakage into the brain parenchyma of the three autonomic areas in adult SHR-S confirmed these previous reports showing in addition that an intact BBB was present in the pre-hypertensive phase. Although we did not measure brain Ang II availability during the establishment of hypertension, it is highly possible that local Ang II (and its downstream pathways) activated by pressure elevation, participate in BBB extravasation. Indeed, we showed previously in chronically hypertensive aortic coarcted rats a marked activation of brain RAS, as confirmed by augmented angiotensinogen and AT1 receptor expression within the NTS and by the blockade of both, accompanied by pressure reduction after losartan treatment (Sangaleti et al., 2004). Another original observation arising from our data was that, different from other autonomic areas, PVN BBB leakage is progressive during the chronic phase of hypertension, as observed in SHR-S from 3 to 5 months. Additionally, we showed that Ang II effect on BBB lesion was more prominent within the PVN, since changes in leakage area and EBA expression following *icv* Ang II were higher and faster than those observed in the NTS and RVLM. Moreover, our data on the absence of FITC leakage in the hypoglossus nucleus reinforces previous observation on the lack of BBB disruption in non-cardiovascular related areas (Biancardi et al., 2014). Therefore, it appears that hypertension damages preferentially the microcirculatory profile within brain autonomic nuclei (as previously described, Yao and May, 2013; Biancardi et al., 2014; Biancardi and Stern, 2016) as well as in hippocampal and cortical areas (Ueno et al., 2009; Pelisch et al., 2013; Mohammadi and Dehghani, 2014; Takeuchi et al., 2015).

A very important observation of the present study was the rapid correction of BBB leakage in autonomic areas of trained SHR, which was normalized after 2 weeks of exercise training. Importantly, the early restoration of BBB integrity in the PVN, NTS, and RVLM of trained SHR occurred simultaneously with the improvement of autonomic control and preceded the significant AP and HR reduction only observed after the 4th week of training. The improvement of autonomic control was confirmed by the decrease of both LF/HF ratio in the heart (due to a marked HF increase, indicative of parasympathetic modulation) and sympathetic vasomotor variability. These responses improved PI variability and abrogated the continuous increase in SAP variability, characteristics of the sedentary SHR. Moreover, strong positive correlations were observed between BBB leakage in the three autonomic areas and SAP variability, LF-SAP, and LF/HF ratio, while PI variability was negatively correlated with BBB leakage. Previous studies have already documented the efficacy of training to improve autonomic control of the circulation in hypertension (Pan et al., 2007; Agarwal et al., 2011; Masson et al., 2014, 2015) and other cardiovascular diseases (Gao et al., 2007; Ichige et al., 2016), but to our knowledge this is the first report showing that training-induced preservation of BBB integrity correlates with improved autonomic control in hypertensive individuals.

We did not measure Ang II content changes in autonomic areas, but we proved its role in training-induced improvement on BBB function since simultaneous *icv* Ang II infusion completely blocked training effects on both FITC leakage and microglia expression within the PVN. This seems to be a local Ang II effect because a very low, subpressor dose (12.5 ng/h) was infused into the lateral ventricle. The present observations confirmed our previous findings in SHR submitted to a similar T protocol that training downregulates RAS within the PVN, NTS, and RVLM (Chaar et al., 2015) showing in addition that microglia inactivation could contribute to the prevention of BBB dysfunction in SHR-T. Previous studies have already demonstrated the relationship between increased brain Ang II availability, augmented reactive oxygen species and pro-inflammatory cytokines, microglial activation and loss of BBB integrity (Shi et al., 2010; Zubcevic et al., 2011; Biancardi et al., 2014). The new finding of the present study is that training, by reducing Ang II availability, is able to interrupt this vicious cycle and preserve BBB integrity even in the presence of hypertension. Additional support for this proposition is our observations on both AT_1_ receptors' downregulation following exogenous Ang II infusion and training-induced AT_1_ receptors' upregulation, which is suggestive of reduced Ang II availability within autonomic areas of the SHR-T. Indeed, in age-matched SHR submitted to a similar training protocol, we have already shown a prompt (2 weeks) downregulation of the RAS within the PVN, NTS, and RVLM simultaneously with reduced sympathetic and increased parasympathetic activity to the heart, decreased vasomotor sympathetic outflow and smaller pressure variability (Felix and Michelini, 2007; Chaar et al., 2015). In trained adult SHR we also showed normalization of autonomic dysfunction and baroreflex sensitivity occurring simultaneously with an early (2 weeks) and marked decrease of p47^phox^ and gp91^phox^ expression, reduced oxidative stress, decreased phosphorylation of ERK1/2, normalization of NF-kB translocation to the nucleus, reduced microglia activation and decreased inflammation within the PVN (Masson et al., 2014, 2015). These observations reinforce the deactivation of Ang II and its downstream pathways by the exercise training. Consistent with those findings, our data on augmented EBA expression within the PVN, NTS, and RVLM confirmed the training-induced improvement of BBB integrity after 2 weeks of exercise, while their reduction in SHR-T simultaneously infused with Ang II validated the involvement of local RAS on BBB effects induced by training. The efficacy of training to improve BBB integrity and ameliorate brain functions was also documented in other pathologies as diabetes, stroke and neurodegenerative diseases (Ding et al., 2006; Zhang et al., 2013; Wang et al., 2014; de Senna et al., 2015; Souza et al., 2016). In addition, trained normotensive rats exhibited small improvements on both BBB integrity and PI variability, reinforcing the importance of an almost intact BBB for normal brain perfusion and adequate neuronal activity that drives the autonomic control of the circulation.

We are aware that commercial AT1 receptor antibodies are non-specific, which could represent a caveat in the interpretation of its immunohistochemistry data. For this reason, we confirmed the involvement of local RAS by comparing BBB permeability in trained SHR simultaneously infused with saline or Ang II *icv*. These functional data do support the role played by the vasoconstrictor, proliferative, pro-inflammatory axis of the brain RAS in damaging (high Ang II content in the SHR-S) or improving (low Ang II availability in SHR-T) the BBB permeability. We also identified training-induced time course and the magnitude of BBB changes within the PVN, NTS, and RVLM, but we did not clarify whether reduced BBB extravasation in exercised SHR was due to changes in paracellular and/or transcellular transport across the endothelium. In addition, our experiments did not reveal the barrier component(s) contributing to hypertension-induced deficits and training-induced benefits observed in sedentary and trained SHR, respectively. Experiments are now being conducted in our laboratory in order to elucidate the mechanism(s) by which aerobic training reverses and corrects BBB permeability in hypertensive individuals. Nevertheless, our data disclose the importance of training to change tissue Ang II content (and microglia activation), whose reduction is crucial for both the maintenance of BBB integrity and the normalization of autonomic control of the circulation in hypertensive individuals.

In conclusion the present set of data uncover a novel benefit of exercise training for hypertensive individuals. This non-pharmacological tool proved to be highly efficient in reversing the deleterious effect of hypertension on BBB leakage within autonomic areas, an effect strongly correlated with the improvement of both parasympathetic and sympathetic control of cardiovascular parameters, even in the persistence of hypertension. Since aerobic training has no side effects, it is a useful adjuvant therapeutic strategy to maintain BBB integrity and adequate tissue perfusion, with clear clinical implications.

## Author contributions

LB: participated in the research design, acquired and interpreted data, wrote the manuscript; MJ: participated in the research design, acquired and interpreted data; MF, AR, and AC: performed the experiments, analyzed, and revised data; LM: conceived, designed and coordinated the research, wrote and revised the manuscript; All authors read and approved the final draft of the manuscript.

### Conflict of interest statement

The authors declare that the research was conducted in the absence of any commercial or financial relationships that could be construed as a potential conflict of interest.
